# Perivascular spaces, diffusion MRI markers and cognitive decline in cerebral small vessel disease

**DOI:** 10.1016/j.cccb.2025.100405

**Published:** 2025-10-24

**Authors:** Gemma Solé-Guardia, Hao Li, Jessica Lebenberg, Mina A Jacob, Ivy Uszynski, Roy P C Kessels, David G Norris, Cyril Poupon, Hugues Chabriat, Frank-Erik de Leeuw, Eric Jouvent, Anil M Tuladhar

**Affiliations:** a Department of Neurology, Research Institute for Medical Innovation, Radboud university medical center, Donders Institute for Brain, Cognition and Behaviour, Centre for medical neuroscience, Nijmegen, the Netherlands; b Department of Medical Imaging, Anatomy, Research Institute for Medical Innovation, Radboud university medical center, Donders Institute for Brain, Cognition and Behaviour, Centre for Medical Neuroscience, preclinical imaging center PRIME, Radboudumc Alzheimer Center, Nijmegen, the Netherlands; c APHP, Lariboisière Hospital, Department of Neurology and CNVT-CERVCO, Paris, France; d Institut du Cerveau et de la Moelle épinière, Paris Brain Instituture, Inserm U1127, CNRS UMR 7225, Sorbonne Université, F-75013, Paris, France; e NeuroSpin, Université Paris-Saclay, CNRS, CEA, Gif-sur-Yvette, France; f Donders Institute for Brain, Cognition and Behaviour, Centre for Cognition, Radboud University, Nijmegen, the Netherlands; g Vincent van Gogh Institute for Psychiatry, Venray, the Netherlands; h Radboudumc Alzheimer Center, Radboud university medical center, Nijmegen, the Netherlands; i Donders Institute for Brain, Cognition and Behaviour, Centre for Cognitive Neuroimaging, Radboud University, Nijmegen, the Netherlands; j Université Paris Cité, FHU NeuroVasc, Paris, France; k APHP, Lariboisière Hospital, Department of Neurology, Paris, France

**Keywords:** Cerebral small vessel disease, Cerebral autosomal dominant arteriopathy with subcortical infarcts and leukoencephalopathy, Perivascular spaces, Diffusion tensor image analysis along the perivascular space index, Peak width of skeletonized mean diffusivity, Dementia

## Abstract

**Background:**

MRI markers, including visible perivascular spaces (PVS), diffusion tensor image analysis along the perivascular space (DTI-ALPS) index, and peak width of skeletonized mean diffusivity (PSMD) may capture the earliest pathogenesis of cerebral small vessel disease (SVD). This study aimed to elucidate the association between these markers and cognitive decline in sporadic and hereditary SVD.

**Methods:**

We included individuals from two cohorts: (1) participants with sporadic SVD from the Radboud University Nijmegen Diffusion tensor Magnetic resonance imaging Cohort (RUNDMC) and (2) participants with cerebral autosomal dominant arteriopathy with subcortical infarcts and leukoencephalopathy (CADASIL) followed at the French National Referral Center. Individuals in both cohorts underwent neuroimaging and cognitive assessment over 14 years. We quantified baseline PVS burden, DTI-ALPS index and PSMD. We used linear mixed models to test their associations with longitudinal cognitive function, and Fine-and-Gray models to assess their association with incident all-cause dementia.

**Results:**

Cohort 1 included 446 individuals (mean age (SD) 65.2 years (8.9); 203 women), Cohort 2 included 164 individuals (mean age (SD) 49.9 years (12.6); 88 women). Baseline DTI-ALPS index was independently associated with better longitudinal processing speed (Cohort 1:β=0.11, 95 %CI 0.03–0.19) and cognitive index (Cohort 2:β=0.20, 95 %CI 0.06–0.33). Neither PVS burden, DTI-ALPS index nor PSMD were significantly associated with increased risk of all-cause dementia.

**Conclusion:**

These findings suggest that DTI-ALPS index may serve as a marker for cognitive decline. However, these markers have limited association with all-cause dementia risk. Future studies are needed to validate DTI-ALPS index and its link to cognitive decline.

## Highlights

• DTI-ALPS index may serve as a marker for cognitive decline.
• DTI-ALPS index may capture localized microstructural abnormalities.
• PVS burden, DTI-ALPS index and PSMD have limited value in predicting dementia risk.

## Introduction

Cerebral small vessel disease (SVD) is recognized as the major vascular contributor to cognitive impairment and dementia [1]. SVD is present in virtually every individual older than 60 and refers to pathological alterations of the smallest brain vasculature in (sporadic) hypertension-related SVD, while a monogenic form affects younger patients in cerebral autosomal dominant arteriopathy with subcortical infarcts and leukoencephalopathy (CADASIL) [2]. Several radiological markers are thought to result from SVD, including white matter hyperintensities (WMH), lacunes, cerebral microbleeds and dilated perivascular spaces (PVS) [3]. Despite advances, accurately predicting cognitive decline as well as dementia onset in individuals with SVD remains challenging.

Among the presumed mechanisms in SVD are glymphatic dysfunction [4] and (microstructural) white matter lesions [5]. The glymphatic system is considered a dynamic waste clearance pathway in the brain involving PVS [6]. There has been growing interest in MRI markers, based on conventional and diffusion MRI, that may reflect these mechanisms [7]. MRI-visible PVS, part of the updated version of standards for reporting vascular changes on neuroimaging (STRIVE) 2.0 criteria [3], have been used to assess impaired waste clearance and have been linked to glymphatic dysfunction [8,9]. Diffusion tensor image analysis along the perivascular space (DTI-ALPS) index, while originally proposed as an indirect proxy of glymphatic function, lacks direct validation and is thought to capture white matter microstructural changes; its interpretation therefore warrants caution [7]. Peak width of skeletonized mean diffusivity (PSMD), a diffusion MRI-derived marker, has been proposed to capture global white matter microstructural changes associated with SVD [10]. Evidence of their association with long-term cognitive decline over an extended follow-up period is still lacking. Prospective studies examining these markers at baseline will clarify their association with disease progression. In particular, examining these markers alongside standard SVD markers such as WMH, lacunes and cerebral microbleeds may help elucidate whether their independent associations with long-term cognitive decline. Integrating sporadic and genetic forms of SVD cohorts may be such an approach to not only accurately characterize whether these markers reflect core disease mechanisms, but also improves generalizability, provides complementary insights, and lays a stronger foundation for accurate risk assessment and development of preventive strategies in a broader context than studying each form in isolation.

In this study, we primarily aimed to examine the associations between these markers of SVD pathology at baseline, including PVS burden and diffusion MRI markers (DTI-ALPS index and PSMD), and long-term cognitive decline in individuals with SVD. Secondly, we aimed to investigate the association between these baseline MRI markers and incident all-cause dementia. For this, we studied baseline measures of these markers in a large longitudinal multi-etiological sample including individuals with sporadic SVD and individuals with CADASIL who have been followed up for over 14 years.

## Materials and methods

### Study population

Individuals with SVD from two independent longitudinal cohorts were included in this study. Details of these cohorts have been already reported [11,12]. Cohort 1 is embedded within the Radboud University Nijmegen Diffusion tensor and Magnetic resonance imaging Cohort (RUN DMC) study (Netherlands), an ongoing cohort study among individuals with sporadic SVD investigating the causes and clinical outcomes of SVD. Inclusion criteria were age between 50 and 85 years and presence of SVD neuroimaging markers (appearance of WMH and/or lacunes). Of the 503 individuals who participated at baseline in 2006, 57 individuals were excluded due to territorial infarcts present on imaging, bringing the final sample size for Cohort 1 to 446. Cohort 2 is embedded within the CADASIL prospective study at the University Hospital Lariboisière, Paris (France). Subjects were recruited at the National Referral Center in Paris, France (https://cervco.fr/) between September 2003 and April 2014. CADASIL diagnosis was confirmed by genetic testing showing a typical sequence variant of NOTCH3 altering the number of cysteine residues. Of the 275 subset of individuals who underwent diffusion MRI between September 2003 and April 2014, 26 individuals were excluded due to presence of dementia at baseline, 79 individuals were excluded due to uncompleted/low quality MRI and 6 individuals were excluded due to failure in imaging pre-processing. This brought the final sample size for Cohort 2 to 164.

In this study, we will refer as “baseline” to the 2006 assessment for Cohort 1 and to the first imaging visit between September 2003 and April 2014 for Cohort 2. Assessments performed after baseline will be referred as “follow-up” for both cohorts (see Supplementary information Table S1 for detailed information on follow-up assessments).

The study was approved by an independent ethics committee at both centers (Cohort 1: Medical Review Ethics Committee Region Arnhem–Nijmegen, reference no CMO 2005–256; Cohort 2: Ethics Committee of Saint Louis Hospital, reference no P020921-AOR02001, Paris). All participants gave written informed consent to participate.

### Neuropsychological assessment

All participants underwent a standardized cognitive assessment battery both at baseline and during follow-up assessments. For Cohort 1, the available neuropsychological test battery has been extensively described elsewhere [13]. Compound scores for global cognitive function (cognitive index), processing speed and executive function were available. Cognitive index was calculated as a compound score using the mean of the z-scores of all tests from the neuropsychological test battery, based on the group mean. Processing speed was calculated as the mean of the z-scores of the 1-letter subtask of the Paper-Pencil Memory Scanning Task, the reading and color naming subtest of the abbreviated Stroop Colour Word Test and the Symbol Digit Modalities Test. Executive function was measured using the semantic fluency task, the interference score of the Stroop Colour Word Test, which was calculated by dividing the performance (in seconds) on the color-word interference card by the mean response time on the reading and color naming cards of the Stroop Colour Word Test, and the Verbal Series Attention Test. For Cohort 2, Mattis Dementia Rating Scale (MDRS) [14] was available as an indicator of global cognitive function. In addition, the raw Trail Making Test (TMT) Part A performance (in seconds) was used as measure of processing speed, and ratio between performance on TMT Part B and Part A (TMT-B/A) [15] was used as an index of executive function.

### Incident all-cause dementia

All-cause dementia case finding is extensively detailed elsewhere [16]. Briefly, participants were first screened with the Mini-Mental State Examination (MMSE) [17] and the Instrumental Activities of Daily Living (IADL) scale [18]. Participants screened positive if they showed an MMSE score below 26 or a decline of ≥3 points compared to the previous assessment, and an IADL below 8. An IADL score below 8 reflects limitations in at least one item of the IADL scale [19]. Individuals who screened positive were subsequently evaluated by an independent multidisciplinary consensus panel meeting consisting of a neurologist, a clinical neuropsychologist and a geriatrician. Dementia diagnosis was adjudicated by evaluating all available clinical data, neuropsychological examinations, depressive symptom scores (assessed with the Center for Epidemiologic Studies Depression Scale [20]). For individuals who did not participate at the follow-up (i.e., those who were deceased or who declined participation), medical records on their cognitive status were retrieved from the general practitioner or nursing home treating physicians. These cases were reviewed again independently by the members of the consensus panel. The diagnosis of dementia was based on the DSM-5 clinical criteria for major neurocognitive disorder due to a neurodegenerative disease.

For individuals in Cohort 2, diagnosis of dementia at baseline was based on the DSM-IV-TR criteria [21]. 26 participants were diagnosed with dementia at baseline based on these criteria. These were excluded from all subsequent analysis. Consensus diagnosis was not available at follow-up for participants in Cohort 2. Therefore, a psychometric approach was used to harmonize our datasets; participants were screened positive retrospectively if they had an MMSE score below 26 or a decline of ≥3 points compared to the previous assessment, and an IADL score below 8. Based on this, 19 participants fulfilled the incident all-cause dementia criteria at baseline. We excluded these 19 cases from our analysis focused on the association between MRI markers and incident all-cause dementia.

The date of all-cause dementia onset was defined as the date of diagnosis. When the date of diagnosis was not exactly known (namely for participants in Cohort 2), we used the date of the follow-up visit when they met the aforementioned criteria.

### MRI acquisition

Only baseline MRI acquisitions were considered in this study.

Participants in Cohort 1 were scanned on a 1.5T MRI scanner (Magnetom Avanto, Simens) with an 8-channel head coil. The imaging protocol included: 3D T1-weighted magnetization-prepared rapid gradient-echo sequence (isotropic voxel size 1.0 mm^3^), T2*-weighted gradient echo sequence (isotropic voxel size 2.5 mm^3^), fluid-attenuated inversion recovery (FLAIR) image (voxel size 0.5  ×  0.5  ×  5.0 mm, interslice gap 1.0 mm), and single-shell diffusion-weighted image (DWI, voxel size = 2.5 × 2.5 × 2.5 mm, 7 vol with *b* = 0 s/mm^2^, 61 vol with *b* = 900 s/mm^2^). Full acquisition details have been described previously [22].

Participants in Cohort 2 were scanned on a 1.5T MRI scanner (Signa, General Electric Medical Systems). The imaging protocol included: 3D T1-weighted sequence (voxel size 1.0  ×  1.0  ×  1.6 mm, interslice gap 0.8 mm), FLAIR image (voxel size 0.5  ×  0.5  ×  5.5 mm, no interslice gap), T2*-weighted gradient-echo sequence (voxel size 0.9  ×  0.9  ×  5.5 mm, no interslice gap), and single-shell diffusion-weighted image (DWI, voxel size = 0.9 × 0.9 × 5.5 mm, interslice gap 1.5 mm, 1 vol with *b* = 0 s/mm^2^, 7–23 vol with *b* = 1000 s/mm^2^). Full acquisition details have been described previously [23].

### MRI markers of SVD

MRI markers of SVD were rated according to the STRIVE 2.0 criteria [3]. In Cohort 1, WMH volumes were calculated by a semiautomatic WMH segmentation method (described in detail elsewhere [24]). Number of lacunes were rated manually on FLAIR and T1-weighted MRI scans, and number of microbleeds were rated manually on T2*-weighted MRI by two trained raters who were blinded to the clinical data [25]. Burden of visible PVS in the basal ganglia (BG-PVS) and in the centrum semiovale (CSO-PVS) were defined as 0 = none, 1 = 1–10, 2 = 10–20, 3 = 21–40, and 4 ≥40 [26]. Intrarater and interrater reliability weighted kappa were excellent (weighted kappa > 0.75) for lacunes, microbleeds [25] and PVS. Brain volume was calculated as the sum of grey matter and white matter volumes. Intracranial volume was calculated as the sum of grey matter, white matter and cerebrospinal fluid volumes.

In Cohort 2, WMH volumes were estimated using Statistical Parametric Mapping (SPM12) (https://www.fil.ion.ucl.ac.uk/spm) lesion segmentation tool, a semiautomatic WMH segmentation method based on lesion prediction algorithm (27), followed by manual correction by a trained scientist to ensure accuracy and consistency [28]. Lacunes were semi-automatically identified and microbleeds were rated manually on T2*-weighted MRI by a trained scientist [29,30]. Burden of visible PVS were rated manually by an experimented neurologist as described on the CADAMRIT inventory [31]. Intrarater and interrater agreements were from good to excellent (weighted kappa > 0.60) for microbleeds [30], lacunes and PVS [31]. Brain and intracranial volumes were calculated using Advanced Normalization Tools (ANTs) [32].

MRI markers of SVD were harmonized as follows: Absolute WMH volume was normalized to each individual’s intracranial cavity (normalized WMH volume=[WMH volume/intracranial volume] × 100) [23,32]. For both cohorts, we extracted presence and count of lacunes and microbleeds. PVS burden was dichotomized into mild (Cohort 1: Scores 0,1; Cohort 2: Score 0) and moderate to severe (Cohort 1: Scores 2–4; Cohort 2: Scores 1,2) categories. Thus, in both cohorts a mild PVS burden reflects <10 PVS, and moderate to severe >10 PVS in the basal ganglia or centrum semiovale.

### Vascular risk factors

Baseline prevalence of hypertension, diabetes, hypercholesterolemia, and smoking was assessed using a standardized questionnaire [22], and a cumulative vascular risk factor (VRF) score (0–4) was derived based on the presence of these factors [33].

### Diffusion MRI (pre-)processing

In both cohorts, diffusion MRI data were preprocessed as described elsewhere [8]. Briefly, preprocessing included denoising, removal of Gibbs artefacts, and correction of head motion, eddy-current-induced distortions, susceptibility-induced distortion (“topup”), and intensity bias, using MRtrix 3.0 software, Functional Magnetic Resonance Imaging of the Brain Software Library (FSL; v6.0.1), Synb0-DISCO and ANTs (ANTs, v 2.1.0) [[34], [35], [36], [37], [38]]. For Cohort 1, where no b0 image with reversed phase encoding was acquired, we generated a synthesized b0 image from the T1 image using Synb0-DISCO to enable “topup” correction [35,36,38]. Processed diffusion data were used to calculate diffusion tensor [36]. In Cohort 2, where no b0 image with reversed phase encoding was acquired, hardware limitations prevented Synb0-DISCO synthesis. As a result, “topup” correction was omitted. Instead, we visually inspected all diffusion images for susceptibility-induced distortions, and those individuals with marked distortions were excluded from further analysis. Additionally, diffusion MRI in Cohort 2 was resliced in the z-direction to align precisely with our regions of interest for DTI-ALPS index calculation. DTI-ALPS index was calculated using a semiautomated method in SVD [39]. Calculation details are described elsewhere [8] and in the Supplementary information. Peak width of skeletonized mean diffusivity (PSMD) was calculated with the PSMD tool provided at http://www.psmd-marker.com [10].

### Statistical analysis

Continuous variables were tested for assumption of normality using Shapiro-Wilk’s test and are presented as mean (standard deviation (SD)) or median (interquartile range (IQR)), with both displayed in Table 1 for completeness. Categorical variables were presented as count and percentage (n, %).

**Table 1:** Baseline Demographic, Cognitive Function and Neuroimaging Characteristics of the SVD samples. Values are shown as mean ± SD; median (IQR). BG-PVS perivascular spaces in basal ganglia, CSO-PVS perivascular spaces in centrum semiovale, DTI-ALPS diffusion tensor image analysis along the perivascular space index, IQR interquartile range, MMSE Mini-Mental State Examination, PSMD peak width of skeletonized mean diffusivity, WMH white matter hyperintensity. ^a^ For Cohort 1, global cognitive function was assessed using a compound score of all tests from the neuropsychological battery referred as cognitive index, processing speed was calculated as the mean of the z-scores of the 1-letter subtask of the Paper-Pencil Memory Scanning Task, the reading and color naming tasks of the Stroop Colour Word Test and the Symbol Digit Modalities Test. Executive function was measured using the verbal fluency task and the interference score of the Stroop Colour Word Test. ^b^ For Cohort 2, the Mattis Dementia Rating Scale was available as an indicator of global cognitive function, raw TMT Part A score was used as indicator of processing speed and ratio between performance on TMT Part B and Part A (TMT-B/A) was used as an index of executive function.

	Cohort 1 Sporadic SVD (*n* = 446)	Cohort 2 CADASIL (*n* = 164)
Demographic		
Age, years	65.2 ± 8.9; 64.3 (57.6,72.6)	49.9 ± 12.6; 49.4 (40.5, 59.5)
Sex, female ( %)	203 (45.5)	88 (53.7)
Education, years	10.9 ± 3.6; 10 [8,15]	11.5 ± 3.8; 11 [9,15]
Cognitive function		
MMSE	28.2 ± 1.6; 29 [27,29]	27.7 ± 2.9; 29 [27,30]
Global cognitive function	0.02 ± 0.73; 0.01 (−0.52, 0.55) ^a^	137.1 ± 9.4; 141 (135, 143) ^b^
Processing speed	0.045 ± 0.85; 0.06 (−0.59, 0.68)^a^	46.8 ± 35.6; 36 (26, 52.8) ^b^
Executive function	0.01 ± 0.75; −0.01 (−0.53, 0.59) ^a^	2.7 ± 1.0; 2.5 (2.0, 3.0)^b^
MRI markers		
Absolute WMH volume, mL	7.9 ± 11.9; 3.1 (1.1,10.0)	79.0 ± 62.2; 60.9 (35.0, 111.6)
Normalized WMH volume ( %)	0.54 ± 0.79; 0.22 (0.08, 0.70)	5.5 ± 4.2; 4.37 (2.38, 7.75)
Lacunes, n ( % of presence)	96 (21.5)	124 (75.6)
Microbleeds, n ( % of presence)	70 (15.7)	48 (29.3)
Brain volume, mL	1068.7 ± 78.0; 1077.0 (1016.0, 1124.1)	1187.1 ± 127.3; 1184.8 (1100.8, 1276.3)
BG-PVS, n moderate to severe ( %)	158 (35.4)	109 (66.5)
CSO-PVS, n moderate to severe ( %)	152 (34.1)	78 (47.6)
DTI-ALPS index	1.44 ± 0.20; 1.44 (1.29, 1.58)	1.22 ± 0.12; 1.21 (1.14, 1.29)
PSMD 10^–3^ mm^2^/s	0.33 ± 0.18; 0.29 (0.25, 0.35)	0.78 ± 0.47; 0.61 (0.43, 1.04)

To examine the association between baseline measures of markers of SVD pathology, PVS burden, DTI-ALPS index and PSMD, and long-term cognitive decline in individuals with SVD over 14 years, we performed linear mixed model analyses following a hierarchical approach. Model 1 included these measures and adjusted for age, sex, education and brain volume. Model 2 additionally adjusted for VRF score, and Model 3 also adjusted for traditional SVD markers (WMH volume, lacunes count, microbleeds count). All models included random effects intercept per participant. Linear mixed model analyses were performed separately per cohort included in this study. For all linear mixed model analyses, the dependent variables that did not meet a normal distribution were transformed using the Yeo-Johnson approach [40]. Multicollinearity was also assessed in all linear mixed model analyses using variance inflation factors. As variance inflation factors was low (<3) in all models, we determined that high multicollinearity, defined as variance inflation factors > 5, was absent [41]. The results of Models 1 and 2 are reported in the Supplementary information (Table S2,S3), whereas the results of Model 3 are reported in the main manuscript. For individuals with sporadic SVD, where normalized grey matter volume was available, we performed linear mixed model analyses adjusted for age, sex, education, and grey matter volume (Model 1), VRF score (Model 2), and additionally traditional SVD markers (WMH volume, lacunes count, microbleeds count; Model 3). These models are reported in the Supplementary Information (Table S4).

To investigate the association between these markers at baseline and incident all-cause dementia, we first calculated person-years at risk from the date of baseline assessment until onset of all-cause dementia, death or date of last follow-up. Cumulative incidence of all-cause dementia risk was calculated using Kaplan-Meier analyses for dichotomized PVS burden, DTI-ALPS index and PSMD. Differences were tested using log-rank tests. Furthermore, we used the proportional hazards model of Fine-and-Gray (FG) [42] to control for age, sex, education, brain volume and death as competing risk (Model 1; Table S5). Model 2 additionally adjusted for VRF score (Table S6), whereas Model 3 also included traditional SVD markers (Table 3). Kaplan-Meier analyses and FG models were performed separately per cohort included in this study. Given that 19 participants from Cohort 2 fulfilled the incident all-cause dementia criteria (assessed retrospectively), we excluded these cases from our analysis focused on the association between MRI markers and incident all-cause dementia. For individuals with sporadic SVD, where normalized grey matter volume was available, FG analyses (Models 1–3) were adjusted for grey matter volume and results are reported in the Supplementary Information (Table S7).

Finally, we examined the correlations among PVS burden, DTI-ALPS index and PSMD with traditional SVD markers using Spearman correlation.

Correction for multiple comparisons was done by FDR correction. All statistical analyses were performed using R software (version 4.4.1) and alpha was set at 0.05 (two-tailed).

## Results

Detailed demographic, cognitive and neuroimaging data are presented in Table 1. We included 446 individuals from Cohort 1 and 164 from Cohort 2. Individuals in Cohort 1 had a mean age of 65.2 (SD 8.9) years at baseline; 203 individuals (45.5 %) were women. Median follow-up time for individuals in Cohort 1 was 9.0 (IQR 6.0 – 13.7) years between baseline and last follow-up. Individuals in Cohort 2 had a mean age of 49.9 (SD 12.6) years at baseline; 88 individuals (53.7 %) were women. Median follow-up time for individuals in cohort 2 was 10.1 (IQR 4.6 – 13.6) years between baseline and last follow-up.

### DTI-ALPS index is associated with cognitive decline

Linear mixed model analyses showed that 1-SD higher DTI-ALPS index at baseline was associated with better cognitive function over time in both cohorts. Specifically, in individuals with sporadic SVD, it was linked to 0.10-SD better performance in processing speed at follow-ups, which showed a statistical trend when adjusted for age, sex, education, VRF score and SVD markers (Cohort 1: standardized β = 0.10, FDR-corrected *p* = 0.05; Table 2). Notably, models excluding SVD markers (Tables S2,S3), and those adjusting for normalized grey matter volume instead of total brain volume (Table S4), did reach significance. In individuals with CADASIL, it was associated with 0.20-SD better global cognitive performance (Cohort 2: standardized β = 0.20, FDR-corrected *p* = 0.01; Table 2). No further significant associations were found between baseline BG-PVS burden, CSO-PVS burden, or PSMD and cognitive function over time in either cohort.

**Table 2:** Association between baseline MRI markers and longitudinal cognitive performance over 14 years. The models were adjusted for age, sex, education level, VRF score and traditional SVD MRI markers, including normalized WMH volume, Lacunes count, Microbleeds count, and brain volume. BG-PVS perivascular spaces in basal ganglia, CI confidence interval, CSO-PVS perivascular spaces in centrum semiovale, DTI-ALPS diffusion tensor image analysis along the perivascular space index, FDR-p false discovery rate corrected p-values, PSMD peak width of skeletonized mean diffusivity, VRF vascular risk factor score. ^a^ For Cohort 1, global cognitive function was assessed using a compound score of all tests from the neuropsychological battery referred as cognitive index, processing speed was calculated as the mean of the z-scores of the 1-letter subtask of the Paper-Pencil Memory Scanning Task, the reading and color naming tasks of the Stroop Test and the Symbol Digit Substitution Task. Executive function was measured using the verbal fluency task and the interference score of the Stroop Test. N for Global cognitive function/Processing speed/Executive function were 444/443/444. For Cohort 2, MDRS was available as an indicator of global cognitive function, raw TMT Part A score was used as indicator of processing speed and ratio between performance on TMT Part B and Part A (TMT-B/A) was used as an index of executive function. N for Global cognitive function/Processing speed/Executive function were 158/154/145.

	Global cognitive function ^a^			Processing speed ^a^			Executive function ^a^		
	Standardized β	95 % CI	FDR-p	Standardized β	95 % CI	FDR-p	Standardized β	95 % CI	FDR-p
**Cohort 1: Sporadic SVD**									
BG-PVS moderate to severe	−0.02	−0.16–0.12	0.78	−0.09	−0.24–0.06	0.27	0.07	−0.08–0.21	0.57
CSO-PVS moderate to severe	0.08	−0.06–0.21	0.55	0.08	−0.06–0.23	0.27	0.06	−0.08–0.20	0.57
DTI-ALPS index	0.09	0.01–0.17	0.10	0.10	0.02–0.19	0.05	0.07	−0.01–0.15	0.40
PSMD	0.01	−0.05–0.08	0.78	0.04	−0.03–0.11	0.27	−0.001	−0.07–0.07	0.98
**Cohort 2: CADASIL**									
BG-PVS moderate to severe	0.12	−0.14–0.37	0.49	0.15	−0.09–0.38	0.29	−0.12	−0.40–0.16	0.79
CSO-PVS moderate to severe	0.01	−0.22–0.24	0.92	−0.11	−0.32–0.10	0.30	−0.04	−0.30–0.22	0.79
DTI-ALPS index	0.20	0.07–0.33	**0.01**	−0.15	−0.27- −0.03	0.06	0.02	−0.13–0.17	0.79
PSMD	−0.26	−0.53–0.008	0.11	0.21	−0.04–0.45	0.19	−0.19	−0.50–0.11	0.79

### PVS burden, DTI-ALPS index and PSMD and the all-cause dementia risk

**Fig. 1:**
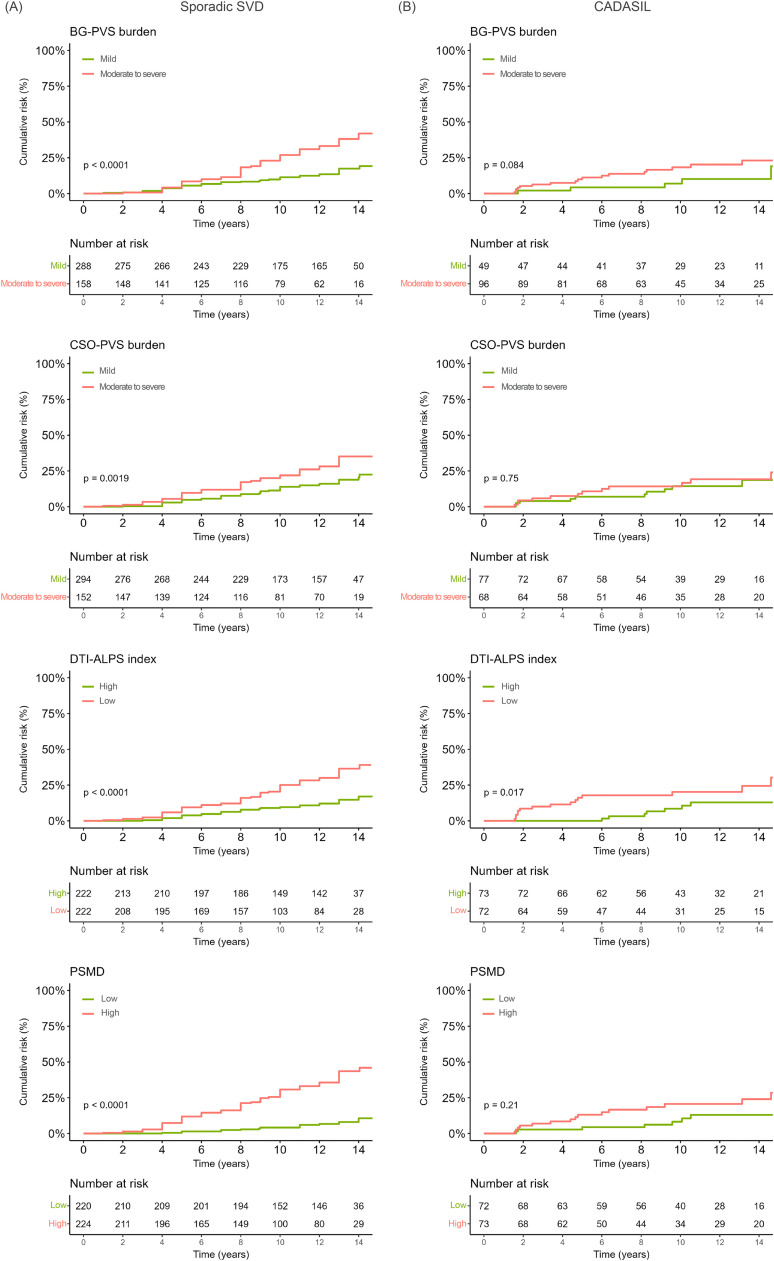
Cumulative risk for all-cause dementia stratified by severity of baseline MRI markers in (A) individuals with sporadic SVD and (B) individuals with CADASIL. BG-PVS perivascular spaces in basal ganglia, CSO-PVS perivascular spaces in centrum semiovale, DTI-ALPS diffusion tensor image analysis along the perivascular space index, PSMD peak width of skeletonized mean diffusivity.

In Cohort 1, 85 out of 446 participants with sporadic SVD (19.1 %) developed dementia over the follow-up period. In Cohort 2, 25 out of 145 participants with CADASIL (17.2 %) developed dementia, after excluding 19 participants who already met the criteria for dementia at baseline. The cumulative risk for all-cause dementia was highest in individuals with sporadic SVD with high BG-PVS and CSO-PVS burden, low DTI-ALPS index and high PSMD values at baseline (Cohort 1; Fig. 1A). In individuals with CADASIL the cumulative risk for all-cause dementia was highest in individuals with low DTI-ALPS index at baseline (Cohort 2; Fig. 1B). After adjustment for age, sex, education, VRF score and SVD markers, neither BG-PVS and CSO-PVS burden, DTI-ALPS index nor PSMD were independently associated with all-cause dementia risk over 14 years in individuals with sporadic SVD or CADASIL, based on FG models accounting for death as a competing risk (Table 3). Fine-and-Gray models adjusted for [1] age, sex, education, and brain volume and [2] age, sex, education, brain volume and VRF score are reported in the Supplementary Information (Table S5, S6).

**Table 3:** Association between baseline MRI markers and incident all-cause dementia in individuals with sporadic SVD and CADASIL over 14 years. The models were adjusted for age, sex, education level, VRF score and SVD MRI markers, including normalized WMH volume, Lacunes count, Microbleeds count, and brain volume. BG-PVS perivascular spaces in basal ganglia, CI confidence interval, CSO-PVS perivascular spaces in centrum semiovale, DTI-ALPS diffusion tensor image analysis along the perivascular space, HR hazard ratio, PSMD peak width of skeletonized mean diffusivity, VRF vascular risk factor score.

	All-Cause Dementia		
	HR	95 % CI	p
**Cohort 1: Sporadic SVD (*N*=444)**			
BG-PVS moderate to severe	1.03	0.67–1.58	0.890
CSO-PVS moderate to severe	1.14	0.77–1.69	0.620
DTI-ALPS index per 1-SD increase	0.97	0.73–1.29	0.850
PSMD (10^–3^ mm^2^/s) per 1-SD increase	0.75	0.40–1.41	0.360
**Cohort 2: CADASIL (*N*=142)**			
BG-PVS moderate to severe	1.37	0.43–4.41	0.600
CSO-PVS moderate to severe	0.93	0.42–2.07	0.860
DTI-ALPS index per 1-SD increase	0.62	0.32–1.21	0.160
PSMD (10^–3^ mm^2^/s) per 1-SD increase	0.75	0.45–1.26	0.270

### Correlations between PVS burden, DTI-ALPS index, PSMD, and traditional SVD MRI markers

**Fig. 2:**
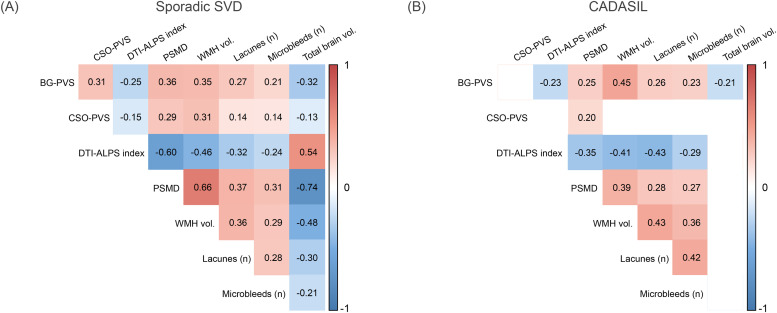
Spearman correlation coefficients between baseline MRI markers in (A) individuals with sporadic SVD and (B) individuals with CADASIL. Coefficients shown are those which false discovery rate corrected p-values reach significance. BG-PVS perivascular spaces in basal ganglia, CSO-PVS perivascular spaces in centrum semiovale, DTI-ALPS diffusion tensor image analysis along the perivascular space index, PSMD peak width of skeletonized mean diffusivity, WMH vol. white matter hyperintensities normalized volume.

In individuals with sporadic SVD, BG-PVS and CSO-PVS burden were positively correlated (Spearman ρ = 0.31, FDR-corrected *p* < 0.001; Fig. 2), and both BG-PVS and CSO-PVS burden further positively correlated with traditional SVD markers (Fig. 2). The DTI-ALPS index was negatively correlated with PVS burden (BG-PVS: Spearman ρ = −0.25, FDR-corrected *p* < 0.001; CSO-PVS: Spearman ρ = −0.15, FDR-corrected *p* = 0.002; Fig. 2), PSMD (Spearman ρ = −0.60, FDR-corrected *p* < 0.001) and traditional SVD markers (Fig. 2). PSMD showed a strong positive association with WMH volume (Spearman ρ = 0.66, FDR-corrected *p* < 0.001). In contrast, in CADASIL, we observed no significant correlations between BG-PVS and CSO-PVS burden, nor between CSO-PVS burden, the DTI-ALPS index and traditional markers of SVD (Fig. 2). Notably, correlations with brain volume and CSO-PVS that were robust in sporadic SVD were largely absent in CADASIL (Fig. 2).

## Discussion

In this large longitudinal data set of an SVD population including individuals with sporadic SVD and individuals with CADASIL, higher DTI-ALPS index at baseline was correlated with better processing speed over time in individuals with sporadic SVD and better global cognitive function (measured as MDRS) in individuals with CADASIL. Baseline measures of PVS burden, DTI-ALPS index and PSMD showed no association with incident all-cause dementia over time after adjusting for baseline standard MRI markers of SVD.

We found that higher baseline DTI-ALPS index was associated with a better performance across multiple cognitive domains over a 14-year follow-up in two large SVD cohorts. Our findings extend previous longitudinal studies in individuals with SVD [8] and Alzheimer’s disease [43,44], by showing that the DTI-ALPS index, measured at baseline, is associated with changes in overall cognitive function and processing speed over time. Notably, these associations remained after adjusting for SVD MRI markers, including PSMD, suggesting that the DTI-ALPS index provides information beyond traditional diffusion metrics of white matter integrity. However, it must be noted that all MRI markers were highly inter-correlated, implying that they may capture a shared SVD burden in both sporadic SVD and CADASIL. While PSMD is well-established and widely used diffusion metric that reflects overall burden of white matter microstructural damage [10], the DTI-ALPS index was originally proposed as an indirect measure of glymphatic function [45]. However, it lacks direct validation, and accumulating evidence suggest that it may offer regional insights into microstructural abnormalities [7]. As its sensitivity to underlying glymphatic dysfunction remains unclear, its interpretation warrants caution. In our analysis, associations between the DTI-ALPS index and cognitive function attenuated to a statistical trend in individuals with sporadic SVD when additionally adjusting for vascular risk factors and SVD hallmarks, yet remained significant in CADASIL. This pattern suggests that the DTI-ALPS index may capture aspects of regional white matter integrity disruption, potentially more pronounced in CADASIL, that are not fully accounted for by global diffusion metrics such as PSMD. To summarize, the DTI-ALPS index’s association with cognitive performance across distinct SVD cohorts supports its potential use as a mechanistic marker of subclinical cognitive impairment, but should not be interpreted as a direct measure of glymphatic dysfunction.

Furthermore, we aimed to establish the association between MRI markers and incident all-cause dementia among a larger and more generalizable SVD sample including sporadic and genetic subtypes (CADASIL). Interestingly, neither PVS burden, DTI-ALPS index nor PSMD were independently associated with incident all-cause dementia in either cohort. The absence of a clear association with incident all-cause dementia contrasts with our findings for continuous cognitive function outcomes. Although dementia is linked to complex multifactorial processes, its diagnosis is ultimately dichotomous, reflecting end stage disease. By the time the diagnostic threshold is reached, widespread pathological processes that extend beyond that of white matter microstructural abnormalities are already present [46]. Instead, the value of these markers may lie in capturing more mechanistic insights in SVD. PVS burden may be indicative of microvascular disruption or impaired clearance pathways, such as glymphatic dysfunction [39]. DTI-ALPS index, while associated with cognitive multiple cognitive domains, may reflect early regional microstructural changes [[47], [48], [49], [50], [51]] that could precede overt neurodegeneration. Likewise, PSMD may not necessarily be directly associated with all-cause dementia onset but global white matter damage [52]. Longitudinal multimodal studies could better elucidate the true incremental value and mechanistic underpinnings of PVS burden, DTI-ALPS index and PSMD in the pathophysiology of SVD.

Major strengths of this study include the inclusion of two deeply phenotyped cohorts, one with sporadic SVD and one with CADASIL, enhancing the generalizability of our findings across different SVD etiologies. The consistent results regarding the association between MRI markers and incident all-cause dementia across both cohorts supports the robustness and reliability of the results. Additional strengths are the inclusion of multiple MRI markers of SVD and the comprehensive longitudinal cognitive data over 14 years. However, several limitations should be acknowledged. First, the disease stage across cohorts is heterogeneous. Individuals with CADASIL (Cohort 2) were, on average, approximately 10 years younger at baseline than those with sporadic SVD (Cohort 1), reflecting the earlier onset in CADASIL [53]. This inherent difference introduces complexity in interpreting diffusion markers, as age and disease stage may affect these in distinct ways across the two groups. Consequently, diffusion abnormalities observed in CADASIL may capture earlier, genetically driven disease mechanisms, whereas in sporadic SVD they may reflect the combined effect of microvascular damage and aging. We addressed this limitation using stratified analysis, but caution is still warranted when interpreting diffusion markers across groups. Second, while the cohorts were not retrospective, all-cause dementia screening was harmonized retrospectively, and different cognitive test batteries were administered across cohorts, which can affect the comparability of cognitive outcomes. This limitation was addressed by focusing on data harmonization and aligning cognitive domains. Furthermore, all statistical analyses were conducted separately by cohort and multiple comparisons were controlled using FDR correction, thereby reducing the risk of confounding and minimizing false-positive findings. Third, the DTI-ALPS index currently lacks formal validation, which limits its interpretability with regards to glymphatic function. Further work using more precise methods for assessing glymphatic function is needed to validate our findings.

In conclusion, our study suggests that the DTI-ALPS index holds potential as complementary mechanistic marker reflecting regional microstructural changes associated with subtle cognitive impairment, rather than direct clinical predictor of all-cause dementia onset. However, the clinical utility of DTI-ALPS index as an earlier diffusion-based marker remains limited without further validation to determine whether it reflects regional white matter abnormalities, glymphatic dysfunction, or other underlying mechanisms relevant to SVD. It is therefore essential to establish the biological significance of DTI-ALPS index in the context of SVD and cognitive decline.

## Funding

This work was supported by the funding of Stichting Alzheimer Nederland and Fondation Vaincre Alzheimer (WE.03–2023–21cb to EJ, GSG and AMT) and Fondation Recherche AVC to EJ. FEdL received a VIDI innovational grant from The Netherlands Organization for Health Research and Development (ZonMw grant 016.126.351). Data collected in the CADASIL Cohort were obtained with the help of a research grant from the Agence Nationale de la Recherche (ANR, RHU TRT_cSVD) and support of Association de Recherche en NEurologie VAsculaire (ARNEVA).

## CRediT authorship contribution statement

**Gemma Solé-Guardia:** Writing – review & editing, Writing – original draft, Methodology, Investigation, Formal analysis, Data curation. **Hao Li:** Writing – review & editing, Methodology, Investigation. **Jessica Lebenberg:** Writing – review & editing, Methodology, Investigation, Data curation. **Mina A Jacob:** Writing – review & editing, Methodology. **Ivy Uszynski:** Writing – review & editing, Methodology. **Roy P C Kessels:** Writing – review & editing, Methodology, Investigation. **David G Norris:** Writing – review & editing, Methodology, Investigation. **Cyril Poupon:** Writing – review & editing, Methodology. **Hugues Chabriat:** Writing – review & editing, Visualization, Supervision, Project administration, Methodology, Investigation, Funding acquisition, Conceptualization. **Frank-Erik de Leeuw:** Writing – review & editing, Visualization, Supervision, Project administration, Methodology, Investigation, Funding acquisition, Conceptualization. **Eric Jouvent:** Writing – review & editing, Visualization, Supervision, Project administration, Methodology, Investigation, Funding acquisition, Conceptualization. **Anil M Tuladhar:** Writing – review & editing, Visualization, Supervision, Project administration, Methodology, Investigation, Funding acquisition, Conceptualization.

## Declaration of competing interest

The authors declare that they have no competing interests.

## Data Availability

The data supporting the findings of this study can be obtained from the corresponding author upon reasonable request by qualified investigators after the approval of relevant regulatory bodies.

## Appendix Supplementary materials

Image, application 1
