# Supplementary material for: Perivascular spaces, diffusion MRI markers and cognitive decline in cerebral small vessel disease
Source: Cereb Circ Cogn Behav. 2025 Oct 24;9:100405. doi: 10.1016/j.cccb.2025.100405 (PMC12597285; doi:10.1016/j.cccb.2025.100405)
Supplement: Supplementary file 1 [file mmc1.docx]

# Supplementary information

## DTI-ALPS Calculation

To calculate the DTI-ALPS index we placed four 5-mm diameter spherical regions of interest in the areas of bilateral projection fibers and association fibers at the level of the lateral ventricular body using an ICBM-DTI-81 white matter atlas (PMID: 18255316). Then, these 4 regions of interest in the ICBM template were registered to the individual's diffusion image using the “fnirt” function within FSL. The positions of registered ROIs were visual inspected. DTI-ALPS index was computed as the ratio between the mean of x-axis diffusivity on the projection fibers (Dxproj) and association fibers (Dxassoc) and the mean of y-axis diffusivity on the projection fibers (Dyproj) and z-axis diffusivity on association fibers (Dzassoc).

**Table S1**

| **Cohort 1: Sporadic SVD** | | **Cohort 2: CADASIL** | |
| --- | --- | --- | --- |
| **Time point** | **N** | **Time point** | **N** |
| Baseline (Total sample) | 503 | Baseline (Total sample) | 275 |
| Baseline (Included sample) | 446 | Baseline (Included sample) | 164 |
| Follow-up 1 | 356 | Follow-up 1 | 137 |
| Follow-up 2 | 316 | Follow-up 2 | 115 |
| Follow-up 3 | 221^a^ | Follow-up 3 | 92 |
|  |  | Follow-up 4 | 78 |
|  |  | Follow-up 5 | 61 |
|  |  | Follow-up 6 | 54 |
|  |  | Follow-up 7 | 49 |
|  |  | Follow-up 8 | 38 |
|  |  | Follow-up 9 | 25 |

Study samples over time. Cohort 1 consisted of 503 individuals at baseline (2006), of whom 57 were excluded due to territorial infarcts present on imaging, resulting in a final sample size of 446. Follow-up assessments were conducted in 2011 (follow-up 1), 2015 (follow-up 2) and 2020 (follow-up 3). ^a^ Cognitive data were collected at all follow-ups, but cognitive index, processing speed and executive function were consistently assessed with the same measures only between 2006 to 2015. Therefore, these data were used for the linear mixed model analyses. Incidence of dementia was evaluated following the same criteria in all time points. Cohort 2 consisted of 275 individuals at baseline, of whom 26 were excluded due to presence of dementia at baseline, 79 were excluded due to uncompleted/low quality MRI and 6 were excluded due to failure in imaging pre-processing. This brought the final sample size for Cohort 2 to 164. Follow-up assessments were conducted every 18-24 months.

**Table S2**

|  | Global cognitive function ^a^ | | | Processing speed ^a^ | | | Executive function ^a^ | | |
| --- | --- | --- | --- | --- | --- | --- | --- | --- | --- |
|  | Standardized β | 95% CI | FDR-p | Standardized β | 95% CI | FDR-p | Standardized β | 95% CI | FDR-p |
| Cohort 1: Sporadic SVD |  |  |  |  |  |  |  |  |  |
| BG-PVS moderate to severe | -0.04 | -0.18-0.10 | 0.76 | -0.13 | -0.28-0.02 | 0.20 | 0.05 | -0.10-0.19 | 0.75 |
| CSO-PVS moderate to severe | 0.04 | -0.10-0.17 | 0.76 | 0.05 | -0.10-0.19 | 0.51 | 0.02 | -0.12-0.16 | 0.75 |
| DTI-ALPS index | 0.11 | 0.04-0.19 | 0.02 | 0.13 | 0.05-0.22 | 0.005 | 0.09 | 0.008-0.16 | 0.12 |
| PSMD | 0.002 | -0.06-0.07 | 0.96 | 0.03 | -0.04-0.10 | 0.51 | -0.02 | -0.08-0.05 | 0.75 |
| Cohort 2: CADASIL |  |  |  |  |  |  |  |  |  |
| BG-PVS moderate to severe | 0.05 | -0.22-0.31 | 0.91 | 0.24 | -0.02-0.49 | 0.09 | -0.12 | -0.40-0.15 | 0.77 |
| CSO-PVS moderate to severe | -0.01 | -0.25-0.22 | 0.91 | -0.06 | -0.28-0.17 | 0.62 | -0.02 | -0.27-0.23 | 0.85 |
| DTI-ALPS index | 0.23 | 0.09-0.37 | 0.005 | -0.19 | -0.32- -0.06 | 0.02 | 0.03 | -0.11-0.18 | 0.85 |
| PSMD | -0.39 | -0.67 - -0.11 | 0.01 | 0.32 | 0.06-0.59 | 0.04 | -0.21 | -0.51-0.08 | 0.63 |

Association between baseline MRI markers and longitudinal cognitive performance over 14 years adjusted for brain volume. The models were adjusted for age, sex, education level, and brain volume. BG-PVS perivascular spaces in basal ganglia, CI confidence interval, CSO-PVS perivascular spaces in centrum semiovale, DTI-ALPS diffusion tensor image analysis along the perivascular space index, FDR-p false discovery rate corrected p-values, PSMD peak width of skeletonized mean diffusivity, VRF vascular risk factor score. ^a^ For Cohort 1, global cognitive function was assessed using a compound score of all tests from the neuropsychological battery referred as cognitive index, processing speed was calculated as the mean of the z-scores of the 1-letter subtask of the Paper-Pencil Memory Scanning Task, the reading and color naming tasks of the Stroop Test and the Symbol Digit Substitution Task. Executive function was measured using the verbal fluency task and the interference score of the Stroop Test. N for Global cognitive function/Processing speed/Executive function were 444/443/444. For Cohort 2, MDRS was available as an indicator of global cognitive function, raw TMT Part A score was used as indicator of processing speed and ratio between performance on TMT Part B and Part A (TMT-B/A) was used as an index of executive function. N for Global cognitive function/Processing speed/Executive function were 161/157/147.

**Table S3**

|  | Global cognitive function ^a^ | | | Processing speed ^a^ | | | Executive function ^a^ | | |
| --- | --- | --- | --- | --- | --- | --- | --- | --- | --- |
|  | Standardized β | 95% CI | FDR-p | Standardized β | 95% CI | FDR-p | Standardized β | 95% CI | FDR-p |
| Cohort 1: Sporadic SVD |  |  |  |  |  |  |  |  |  |
| BG-PVS moderate to severe | -0.04 | -0.18-0.10 | 0.76 | -0.12 | -0.27-0.03 | 0.24 | 0.05 | -0.09-0.20 | 0.75 |
| CSO-PVS moderate to severe | 0.04 | -0.10-0.17 | 0.76 | 0.05 | -0.10-0.19 | 0.52 | 0.02 | -0.12-0.16 | 0.75 |
| DTI-ALPS index | 0.11 | 0.04-0.19 | 0.02 | 0.13 | 0.05-0.21 | 0.006 | 0.08 | 0.006-0.16 | 0.14 |
| PSMD | 0.002 | -0.06-0.07 | 0.95 | 0.03 | -0.04-0.10 | 0.49 | -0.02 | -0.08-0.05 | 0.75 |
| Cohort 2: CADASIL |  |  |  |  |  |  |  |  |  |
| BG-PVS moderate to severe | 0.04 | -0.22-0.30 | 0.88 | 0.25 | -0.002-0.50 | 0.07 | -0.13 | -0.40-0.15 | 0.75 |
| CSO-PVS moderate to severe | -0.02 | -0.25-0.30 | 0.88 | -0.05 | -0.27-0.17 | 0.66 | -0.03 | -0.28-0.23 | 0.84 |
| DTI-ALPS index | 0.23 | 0.09-0.37 | 0.004 | -0.19 | -0.32- -0.06 | 0.02 | 0.03 | -0.11-0.18 | 0.84 |
| PSMD | -0.37 | -0.65- -0.09 | 0.02 | 0.30 | 0.03-0.56 | 0.06 | -0.21 | -0.51-0.09 | 0.66 |

Association between baseline MRI markers and longitudinal cognitive performance over 14 years adjusted for VRF score and brain volume. The models were adjusted for age, sex, education level, VRF score, and brain volume. BG-PVS perivascular spaces in basal ganglia, CI confidence interval, CSO-PVS perivascular spaces in centrum semiovale, DTI-ALPS diffusion tensor image analysis along the perivascular space index, FDR-p false discovery rate corrected p-values, PSMD peak width of skeletonized mean diffusivity, VRF vascular risk factor score. ^a^ For Cohort 1, global cognitive function was assessed using a compound score of all tests from the neuropsychological battery referred as cognitive index, processing speed was calculated as the mean of the z-scores of the 1-letter subtask of the Paper-Pencil Memory Scanning Task, the reading and color naming tasks of the Stroop Test and the Symbol Digit Substitution Task. Executive function was measured using the verbal fluency task and the interference score of the Stroop Test. N for Global cognitive function/Processing speed/Executive function were 444/443/444. For Cohort 2, MDRS was available as an indicator of global cognitive function, raw TMT Part A score was used as indicator of processing speed and ratio between performance on TMT Part B and Part A (TMT-B/A) was used as an index of executive function. N for Global cognitive function/Processing speed/Executive function were 161/157/147.

**Table S4**

|  | Global cognitive function ^a^ | | | Processing speed ^a^ | | | Executive function ^a^ | | |
| --- | --- | --- | --- | --- | --- | --- | --- | --- | --- |
|  | Standardized β | 95% CI | FDR-p | Standardized β | 95% CI | FDR-p | Standardized β | 95% CI | FDR-p |
| Cohort 1: Sporadic SVD |  |  |  |  |  |  |  |  |  |
| Model 1 |  |  |  |  |  |  |  |  |  |
| BG-PVS moderate to severe | -0.06 | -0.20-0.09 | 0.72 | -0.14 | -0.29-0.01 | 0.14 | 0.04 | -0.10-0.19 | 0.75 |
| CSO-PVS moderate to severe | 0.04 | -0.09-0.18 | 0.72 | 0.05 | -0.09-0.20 | 0.56 | 0.02 | -0.12-0.16 | 0.75 |
| DTI-ALPS index | 0.14 | 0.07-0.22 | <0.001 | 0.16 | 0.09-0.24 | <0.001 | 0.10 | 0.03-0.18 | 0.03 |
| PSMD | -0.006 | -0.07-0.06 | 0.86 | 0.02 | -0.05-0.09 | 0.56 | -0.02 | -0.09-0.05 | 0.75 |
| Model 2 |  |  |  |  |  |  |  |  |  |
| BG-PVS moderate to severe | -0.05 | -0.20-0.09 | 0.72 | -0.13 | -0.29-0.02 | 0.17 | 0.05 | -0.10-0.19 | 0.76 |
| CSO-PVS moderate to severe | 0.04 | -0.10-0.17 | 0.72 | 0.05 | -0.09-0.20 | 0.49 | 0.02 | -0.12-0.16 | 0.76 |
| DTI-ALPS index | 0.14 | 0.07-0.22 | <0.001 | 0.16 | 0.08-0.24 | <0.001 | 0.10 | 0.03-0.18 | 0.04 |
| PSMD | -0.005 | -0.07-0.06 | 0.88 | 0.03 | -0.04-0.09 | 0.49 | -0.02 | -0.09-0.05 | 0.76 |
| Model 3 |  |  |  |  |  |  |  |  |  |
| BG-PVS moderate to severe | -0.03 | -0.17-0.12 | 0.78 | -0.10 | -0.25-0.06 | 0.31 | 0.06 | -0.09-0.21 | 0.56 |
| CSO-PVS moderate to severe | 0.08 | -0.05-0.22 | 0.47 | 0.09 | -0.06-0.24 | 0.31 | 0.06 | -0.08-0.20 | 0.56 |
| DTI-ALPS index | 0.11 | 0.03-0.08 | 0.02 | 0.13 | 0.05-0.19 | 0.009 | 0.08 | 0.001-0.16 | 0.19 |
| PSMD | 0.010 | -0.06-0.07 | 0.78 | 0.04 | -0.03-0.11 | 0.31 | -0.003 | -0.07-0.07 | 0.94 |

Association between baseline MRI markers and longitudinal cognitive performance over 14 years. All models were adjusted for age, sex, education level. Model 1 was additionally adjusted for grey matter volume, Model 2 was adjusted for grey matter volume and VRF score, and Model 3 was adjusted for grey matter volume, VRF score and SVD MRI markers, including normalized WMH volume, Lacunes count, Microbleeds count. BG-PVS perivascular spaces in basal ganglia, CI confidence interval, CSO-PVS perivascular spaces in centrum semiovale, DTI-ALPS diffusion tensor image analysis along the perivascular space index, FDR-p false discovery rate corrected p-values, PSMD peak width of skeletonized mean diffusivity, VRF vascular risk factor score. ^a^ For Cohort 1, global cognitive function was assessed using a compound score of all tests from the neuropsychological battery referred as cognitive index, processing speed was calculated as the mean of the z-scores of the 1-letter subtask of the Paper-Pencil Memory Scanning Task, the reading and color naming tasks of the Stroop Test and the Symbol Digit Substitution Task. Executive function was measured using the verbal fluency task and the interference score of the Stroop Test. N for Global cognitive function/Processing speed/Executive function were 444/443/444.

**Table S5**

|  | All-Cause Dementia: Model 1 | | |
| --- | --- | --- | --- |
|  | HR | 95% CI | p |
| Cohort 1: Sporadic SVD (N= 444) |  |  |  |
| BG-PVS moderate to severe | 1.03 | 0.67-1.59 | 0.890 |
| CSO-PVS moderate to severe | 1.20 | 0.84-1.72 | 0.320 |
| DTI-ALPS index per 1-SD increase | 0.93 | 0.71-1.23 | 0.630 |
| PSMD (10^–3^ mm^2^/s) per 1-SD increase | 0.94 | 0.75-1.16 | 0.550 |
| Cohort 2: CADASIL (N= 144) |  |  |  |
| BG-PVS moderate to severe | 1.41 | 0.69-2.86 | 0.350 |
| CSO-PVS moderate to severe | 0.76 | 0.38-1.50 | 0.420 |
| DTI-ALPS index per 1-SD increase | 0.62 | 0.36-1.07 | 0.089 |
| PSMD (10^–3^ mm^2^/s) per 1-SD increase | 0.94 | 0.63-1.38 | 0.740 |

Association between baseline MRI markers and incident all-cause dementia in individuals with sporadic SVD and CADASIL over 14 years adjusted for brain volume. The models were adjusted for age, sex, education level, and brain volume. BG-PVS perivascular spaces in basal ganglia, CI confidence interval, CSO-PVS perivascular spaces in centrum semiovale, DTI-ALPS diffusion tensor image analysis along the perivascular space, HR hazard ratio, PSMD peak width of skeletonized mean diffusivity.

**Table S6**

|  | All-Cause Dementia: Model 1 | | |
| --- | --- | --- | --- |
|  | HR | 95% CI | p |
| Cohort 1: Sporadic SVD (N= 444) |  |  |  |
| BG-PVS moderate to severe | 1.03 | 0.67-1.60 | 0.890 |
| CSO-PVS moderate to severe | 1.20 | 0.83-1.73 | 0.330 |
| DTI-ALPS index per 1-SD increase | 0.93 | 0.71-1.23 | 0.630 |
| PSMD (10^–3^ mm^2^/s) per 1-SD increase | 0.94 | 0.75-1.16 | 0.550 |
| Cohort 2: CADASIL (N= 144) |  |  |  |
| BG-PVS moderate to severe | 1.93 | 0.66-5.69 | 0.230 |
| CSO-PVS moderate to severe | 1.00 | 0.45-2.23 | 1.000 |
| DTI-ALPS index per 1-SD increase | 0.50 | 0.28-0.87 | 0.015^a^ |
| PSMD (10^–3^ mm^2^/s) per 1-SD increase | 0.78 | 0.48-1.27 | 0.320 |

Association between baseline MRI markers and incident all-cause dementia in individuals with sporadic SVD and CADASIL over 14 years adjusted for VRF score and brain volume. The models were adjusted for age, sex, education level, VRF score and brain volume. ^a^False discovery rate corrected p-value is 0.06. BG-PVS perivascular spaces in basal ganglia, CI confidence interval, CSO-PVS perivascular spaces in centrum semiovale, DTI-ALPS diffusion tensor image analysis along the perivascular space, HR hazard ratio, PSMD peak width of skeletonized mean diffusivity, VRF vascular risk factor score.

**Table S7**

|  | All-Cause Dementia  Sporadic SVD (N= 444) | | |
| --- | --- | --- | --- |
|  | HR | 95% CI | p |
| Model 1 |  |  |  |
| BG-PVS moderate to severe | 1.07 | 0.70-1.63 | 0.764 |
| CSO-PVS moderate to severe | 1.16 | 0.81-1.66 | 0.414 |
| DTI-ALPS index per 1-SD increase | 0.86 | 0.66-1.13 | 0.290 |
| PSMD (10^–3^ mm^2^/s) per 1-SD increase | 0.97 | 0.82-1.15 | 0.719 |
| Model 2 |  |  |  |
| BG-PVS moderate to severe | 1.07 | 0.70-1.64 | 0.764 |
| CSO-PVS moderate to severe | 1.16 | 0.81-1.67 | 0.418 |
| DTI-ALPS index per 1-SD increase | 0.86 | 0.66-1.13 | 0.290 |
| PSMD (10^–3^ mm^2^/s) per 1-SD increase | 0.97 | 0.82-1.15 | 0.723 |
| Model 3 |  |  |  |
| BG-PVS moderate to severe | 1.05 | 0.69-1.61 | 0.814 |
| CSO-PVS moderate to severe | 1.10 | 0.74-1.62 | 0.649 |
| DTI-ALPS index per 1-SD increase | 0.90 | 0.69-1.18 | 0.457 |
| PSMD (10^–3^ mm^2^/s) per 1-SD increase | 0.86 | 0.64-1.15 | 0.311 |

Association between baseline MRI markers and incident all-cause dementia in individuals with sporadic SVD and CADASIL over 14 years. All models were adjusted for age, sex, education level. Model 1 was additionally adjusted for grey matter volume, Model 2 was adjusted for grey matter volume and VRF score, and Model 3 was adjusted for grey matter volume, VRF score and SVD MRI markers, including normalized WMH volume, Lacunes count, Microbleeds count. BG-PVS perivascular spaces in basal ganglia, CI confidence interval, CSO-PVS perivascular spaces in centrum semiovale, DTI-ALPS diffusion tensor image analysis along the perivascular space, HR hazard ratio, PSMD peak width of skeletonized mean diffusivity, VRF vascular risk factor score.
